# Applying Sutureless Encircling Number 41 Band and Transscleral Chandelier-Assisted Laser Retinopexy for Scleral Buckling Procedure

**DOI:** 10.1155/2017/4671305

**Published:** 2017-11-30

**Authors:** Amir Ramadan Gomaa, Samir Mohamed Elbaha

**Affiliations:** Faculty of Medicine, Alexandria University, Alexandria, Egypt

## Abstract

**Purpose:**

To assess the outcome of sutureless encirlcing number 41 band and transscleral laser retinopexy in uncomplicated rhegmatogenous retinal detachment (RRD), using a wide-angle viewing system (WAVS) and chandelier endoillumination.

**Methods:**

Prospective intervention study included 30 eyes of 30 patients presenting with RRD of recent onset indicated for SB. All cases were treated by sutureless encircling number 41 band and transscleral laser retinopexy. Visualization was provided by the Resight WAVS and a single 27-gauge chandelier endoillumination. Anatomical and visual outcomes were evaluated.

**Results:**

The mean age of our group was 49.8 ± 12.3 years, and the mean duration of RD was 7 (0–50) days. Twenty-four eyes (80.0%) were phakic while the remaining 6 eyes (20%) were either pseudophakic or aphakic. The primary retinal reattachment rate was 83.3% (25 out of 30 eyes). LogMAR visual acuity improved from 1.3 (0.30–2.0) preoperatively to 1.0 (0.40–1.60) at first month (*p* = 0.002) and to 0.70 (0.20–1.92) at third month (*p* < 0.001).

**Conclusion:**

Sutureless encircling number 41 band with chandelier-assisted transscleral laser retinopexy is a safe and effective technique for managing uncomplicated RRD. It provides a high primary success rate while eliminating the complications of cryotherapy, sutures, and broad buckles.

## 1. Introduction

Applying either scleral buckling (SB) or pars plana vitrectomy (PPV) results in comparable success rates in eyes with rhegmatogenous retinal detachment (RRD) and low-grade proliferative vitreoretinopathy (PVR). In pseudophakic eyes, the single-operation success rate is slightly higher with PPV, while in phakic eyes, scleral buckling is superior [1–4]. Despite these established results, SB is declining in popularity while PPV is increasingly favored. This is probably related to the progressive advancements in the technology of PPV together with the decreased training in SB [5].

Nevertheless, SB remains one of the primary methods of RRD repair, because of its major advantage of being an extraocular procedure. This makes it more cost-effective and the better choice in young myopes with adherent posterior hyaloid [6, 7].

Although ophthalmologists are accustomed to the inverted small view of the indirect ophthalmoscopy, localizing small breaks can be difficult in cases with a small pupil, opaque posterior capsular, or media opacities [8–10]. PPV minimizes the chances of missing retinal tears by combining bright endoillumination with wide-angle viewing system (WAVS) and high microscopic magnification. This is unfortunately coupled with an increased risk of cataract, iatrogenic tears, and delayed visual recovery [4, 11]. Trying to seize the benefit of both procedures, some surgeons have described using chandelier endoillumination and contact or noncontact wide-angle viewing systems (WAVS) for scleral buckling with encouraging results [9, 10].

RRD in eyes with retinal breaks in multiple quadrants, diffuse vitreo-retinal pathology or early PVR, are better managed by encircling techniques. Using narrow bands in these cases, instead of wide tires, can reduce orbital tissue manipulation and decrease the risk of extraocular muscle restrictions [12, 13].

By refining the technique of scleral buckling, we could decrease the incidence of complications while maintaining a high primary success rate.

Sutureless scleral buckling removes the risk of inadvertent globe penetration and provides a more regular buckle [14]. Sternberg et al. described this technique as early as 1988, but they used tissue adhesives instead of scleral tunnels [15].

The risk of PVR progression and cystoid macular edema after classical scleral buckling is increased by intraocular inflammation induced by cryotherapy [16].

Haller et al. had shown the effectiveness and safety of tansscleral laser retinopexy for scleral buckling, which could theoretically remove these risks [17].

Our study aimed to assess the outcome of sutureless encircling number 41 band and transscleral laser retinopexy in uncomplicated retinal detachment, using a wide-angle viewing system and chandelier endoillumination.

## 2. Methods

Our prospective noncomparative intervention study was done in the period between March 2014 and November 2015, and it included 30 eyes of patients presenting with RRD of recent onset having peripheral break or breaks. Exclusion criteria included eyes with media opacities, such as vitreous hemorrhage or significant cataract, PVR grade C or D, giant retinal tear, posterior tears, macular hole retinal detachment, and previous retinal detachment surgery. We also excluded children, highly myopic eyes, and eyes with thin sclera to allow for safe sutureless technique.

The university ethics committee approved our study, and informed consent was obtained from all participants and we adhered to the tenets of the declaration of Helsinki.

Complete preoperative evaluation was done for all patients including Snellen best-corrected visual acuity measurement (which was converted to logMAR acuity for analysis), anterior segment slit-lamp examination, Goldmann applanation tonometry, and fundus examination with slit-lamp biomicroscopy and indirect ophthalmoscopy to determine the extent of the detachment, the grade of PVR, and retinal break location and number.

All operations were done under general anesthesia and by the same surgeon (S.E.). In all patients, a 360° limbal peritomy was done followed by passing traction sutures under the four rectus muscles. Scleral tunnels were then made, one in each quadrant, by first making two partial thickness radial incisions with a diamond blade, 3 mm long and 3 mm apart and posterior enough to support most breaks. The scleral tunnels were completed with a crescent knife then number 41 band was passed around the globe and through the tunnels. The end of the band was secured by a Watzke sleeve without tightening. An oblique sclerotomy with a 27-gauge trocar cannula was made 180° away from the primary break and 4 mm from the limbus. A single 27-gauge chandelier light source (disposable Eckard TwinLight Chandelier; DORC International, the Netherlands) was inserted into the trocar, and a noncontact wide-angle viewing system (Resight; Carl Zeiss Meditec, Germany) was applied. Paracentesis was done followed by scleral indentation by the diode laser retinopexy probe (Iris Medical DioPexy; Iridex) to localize the breaks and apply laser to them. Treatment endpoint was a light-gray retinal reaction. Subretinal fluid was externally drained with a 26-gauge needle in indicated cases. The fundus was rechecked with the WAVS to confirm the indentation effect, and the band was tightened through the Watzke sleeve. Finally, the chandelier light and cannula were removed and the insertion site checked for vitreous leak. Scissors were used to excise the vitreous, and 8-0 vicryl was used for conjunctival closure.

Patients were followed up postoperatively at first day, first week, first month, and monthly for 3 months. During each visit, a detailed ophthalmic examination was carried out and the anatomic and functional status of the retina were assessed. Also, complications were recorded and the schedule of follow-up was modified according to the presenting complication.

### 2.1. Statistical Analysis of the Data

Data were fed to the computer and analyzed using IBM SPSS software package version 20.0 (Armonk, NY: IBM Corp). The Kolmogorov-Smirnov, Shapiro, and D'Agostino tests were used to verify the normality of distribution of variables. Comparisons between groups for categorical variables were assessed using chi-square test (Fisher or Monte Carlo). Wilcoxon signed rank test was assessed for comparison between different periods for abnormally distributed quantitative variables. Significance of the obtained results was judged at the 5% level.

## 3. Results

The demographic and clinical characteristics of the patients are presented in Table 1. The mean age of our group was 49.83 ± 12.31 years, and the mean duration of RD was 7 days (0–50). Mean preoperative visual acuity in logMAR units was 1.3 (0.30–2.0). Twenty-four eyes were phakic, 2 aphakic, and 4 pseudophakic. The most prevalent pattern of RD was superior, present in 12 eyes followed by inferior in 10 eyes, temporal in 5 eyes, and total in 3 eyes. Six of the superior RD cases were bullous, and the macula was detached in 19 eyes. Eighteen eyes had single break and 12 had multiple breaks, and the median number of breaks was 1 (1–5). All breaks were peripheral, and their type was either horseshoe tear, atrophic hole, or a mixture of both.

Distribution of the studied cases according to operative details is presented in Table 2. Drainage of subretinal fluid was done in 14 eyes. Air was injected in 8 eyes, and SF_6_ gas in 13 eyes. Three eyes had vitreous leak from the chandelier insertion site, which was excised by scissors without suturing.

Best-corrected visual acuity in logMAR units significantly improved from 1.3 (0.30–2.0) preoperatively to 1.0 (0.40–1.60) at the first postoperative month (*p* = 0.002) and to 0.70 (0.20–1.92) at the third month (*p* < 0.001) (Table 3).

The primary successful retinal reattachment rate was 83.3% (25 out of 30 eyes) while the final success rate was 100%. Of the 5 failed cases, 3 eyes had persistent detachment due to nonclosure of the break and 2 eyes due to progression of PVR. In all 5 eyes, successful retinal reattachment was achieved after pars plana vitrectomy and silicone oil injection.

Further comparison was done between perioperative details in successful and failed cases. No association was found with any variable including lens status, RD duration, RD pattern, drainage of subretinal fluid, or gas injection (Table 4).

## 4. Discussion

In RRD, inability to detect retinal breaks is a prognostic factor for poor surgical outcome. This was the trigger for trying WAVS and chandelier endoillumination in an attempt to improve visualization [18, 19].

Many studies on chandelier-assisted buckling had reported the capability of WAVS-equipped surgical microscope to replace the indirect ophthalmoscope and with better visualization potentials [19, 20]. Although similar observations were noted in our group, buckling under the microscope still had its challenges. Jahangir et al. [21] reported difficulty in rotating the globe with the chandelier light in place. Also, Seider et al. [22] noted that the sutures around the muscles did not provide the same control provided with instruments within sclerotomies in PPV.

In our study, we used the noncontact Resight system for wide fundus viewing. Similar panoramic lenses were used in other studies, either noncontact as BIOM or contact as Mini Quad [18, 20, 23]. Other groups reported satisfactory peripheral examination with the narrow field lenses, as Landers 50° contact prism lens, when combined with scleral compression [19, 24]. However, we preferred the noncontact types due to their easier manipulation and more stable image.

For endoillumination, we used one tip of the 27-gauge chandelier twin light and vitreous leak occurred in only 3 eyes after removing the trocar. Smaller sclerotomies are theoretically associated with less vitreous leak and less risk of infective endophthalmitis [23]. Nevertheless, no case of endophthalmitis has been reported using any of the 25-gauge chandelier systems.

Chandelier insertion may increase the risk of cataract from lens touch and new breaks from vitreous traction during eye manipulation [24, 25]. In most studies [24, 26, 27] including ours, none of these complications occurred. Only Imai et al.'s study [25] reported a new break at the site of the cannula in one eye and lens touch by the endoilluminator during cryoretinopexy in another eye. Fixing the silicone band to the sclera before inserting the chandelier trocar and cannula could minimize the risk of these complications. This sequence was used by Jahangir et al. [21] and Wang et al. [28] and also by our group.

For our cases, we inserted the chandelier 180° from the primary tear. In different studies, the site of endoillumination insertion was determined by surgeon's preference. Some surgeons placed the chandelier either 90°, 120°, or 180° away from the tear [20–22]. Others limit insertion to one of 2 locations, still keeping the light away from the break. The preferred sites were 12 and 6 [23], 5 and 7 [24, 26], or 11 and 1 o'clock positions [27]. Practically, all sites were convenient, as only a slight change in the angle of the chandelier, irrespective of its location, was sufficient to visualize any part of the retina [18, 24]. In our study, a special situation occurred when a tear was very close to the chandelier as the glare from the light source hindered visualization. This condition was managed by either decreasing the intensity of the illumination or slight withdrawal of the chandelier fiber optic.

In our study, we used transscleral diode laser retinopexy instead of cryotherapy, as it is reported to induce less pain and less inflammation. Other advantages included the efficient transmission of the laser through both the band and the sclera and the aiming beam which allow exact localization of the point of indentation [15, 29]. Haller et al. were the first to report the effectiveness and safety of diopexy with only minor complications. The absence of scleral thermal effect in our cases could be due to the exclusion of eyes with thin sclera. Also, using minimal power and duration together with the integrated optic at the tip of the laser probe allowed avoiding Brüch's membrane rupture and hemorrhages. In contrast, older studies reported few cases of Brüch's membrane rupture [30].

Our cases were good candidates for encircling procedure due to the peripheral location of the breaks. The rational of encircling is to relieve vitreoretinal traction by indenting the globe all around thus preventing the intrusion of vitreous through the breaks [31, 32]. Wide buckles are more difficult to apply and are associated with increased risk of infection, extrusion, and reduced ocular blood flow. In contrast, narrow bands are much simpler and faster to place and cause less distortion of the globe contour [33–35]. Furthermore, Banaee et al. [36] proposed that a partial internal tamponade can be created by a narrow band, without the need for wide indentation. Their results actually showed a comparable success rate between the narrow band technique and the wide encircling buckling procedure.

The narrowest silicone band is number 240 (2.5 mm) followed by number 41 (3.5 mm) then number 42 which is the thickest and widest (4.0 mm) [37]. In our study, we used number 41 band (Mira Inc., Uxbridge, MA, USA) in all eyes and none needed augmentation by a sponge or a tire. Few chandelier-assisted SB studies had similarly reported using bands as Seider et al. [22] who used number 42 band in 58% of their cases and Jahangir et al. [21].

Sutureless fixation of the band to the sclera was applied in all our cases. Using this technique necessitated excluding eyes with thin sclera. Nevertheless, the risk of accidental globe penetration with the sutures was removed and the risk of buckle infection, which is related to the use of sutures, was reduced [38, 39].

Mazinani et al. had shown that surgical experience has a significant effect on the success of RD repair surgery, regardless of the technique in question [40]. To address this source of bias, comparative studies were constructed in which the same surgeon used both conventional SB and chandelier-assisted SB techniques. In each of the 3 available studies, both treatment groups achieved an almost similar success rate [22, 23, 39]. The new finding was the shorter duration of buckling in the chandelier group [22, 23].

In our study, the primary success rate was 83.3% (25 out of 30 eyes) which was comparable to previous reports. The reported overall success rate with conventional SB ranged from 77% to 93% [41] and with chandelier-assisted buckling ranged from 83.3% to 95.6% [21, 25–27, 42, 43]. The primary objective of our study was to reduce the sources of complication while maintaining the high success rate, which was actually achieved. We should also take into consideration the learning curve for mastering new techniques. More experience with the new steps could result in even higher success rates.

There were some limitations in our study as the lack of a control group, the relatively short follow-up period, the small number of patients, and the dependence on individual skills of a single surgeon. A randomized comparative study on a larger population with a longer follow-up period is needed to determine if our technique is superior to the traditional SB and to further evaluate its safety.

In conclusion, sutureless encircling band with chandelier-assisted transscleral laser retinopexy is a safe and effective technique for managing uncomplicated RRD. It provides a high primary success rate while eliminating the complications of cryotherapy, sutures, and broad buckles.

## Figures and Tables

**Table 1:** Distribution of the studied cases according to demographic and clinical characteristics.

	Number (%)
*Sex*	
Male	20 (66.7%)
Female	10 (33.3%)
*Eye*	
OD	10 (33.3%)
OS	20 (66.7%)
*Lens*	
Aphakic	2 (6.7%)
Phakic	24 (80.0%)
Pseudophakic	4 (13.3%)
*Break location*	
Superior or supero-temporal	12 (40%)
Temporal	5 (16.7%)
Superior and inferior	3 (10.0%)
Inferior	10 (33.3%)
*Macula*	
Off	19 (63.3%)
On	11 (36.7%)

**Table 2:** Distribution of the studied cases according to operative details.

	Number (%)
*Drainage*	
No	16 (53.3%)
Yes	14 (46.7%)
*Gas type*	
Air	8 (26.7%)
No	9 (30.0%)
SF_6_	13 (43.3%)
*Failure of primary surgery*	
No	25 (83.3%)
Yes	5 (16.6%)

**Table 3:** LogMar visual acuity change.

	Pre	1 m FU	3 m FU
VA lgm	1.3 (0.3–2.0)	1.0 (0.4–1.6)	0.7 (0.2–1.9)
	*p*_1_ = 0.002^∗^, *p*_2_ < 0.001^∗^, *p*_3_ = 0.022^∗^		

Abnormally distributed data was expressed in median (min.–max.). *p*_1_: *p* value for comparing between pre and 1 m FU. *p*_2_: *p* value for comparing between pre and 3 m FU. *p*_3_: *p* value for comparing between 1 m FU and 3 m FU. ^∗^Statistically significant at *p* ≤ 0.05.

**Table 4:** Relation between anatomical success and periopertaive data.

	Success		*P*
	Yes (*n* = 25)	No (*n* = 5)	
Duration (days)	7.0 (0.0–30.0)	10.0 (4.0–50.0)	0.138
Break number	1.0 (1.0–5.0)	1.0 (1.0–4.0)	0.669
*Lens*			
Aphakia	2 (8%)	0 (0.0%)	1.000
Phakic	19 (76%)	5 (100.0%)	
Pciol	4 (16%)	0 (0.0%)	
*Break location*			
Superior or supero-temporal	11 (44%)	1 (20%)	0.616
Temporal	4 (16%)	1 (20%)	
Superior and inferior	2 (8%)	1 (20%)	
Inferior	8 (32%)	2 (40%)	
*Drainage*			
No	13 (52.0%)	3 (60.0%)	1.000
Yes	12 (48.0%)	2 (40.0%)	
*Gas type*			
Air	7 (28%)	1 (20.0%)	0.834
No	8 (32%)	1 (20.0%)	
SF_6_	10 (40%)	3 (60.0%)	

Qualitative data were described using number and percent and were compared using chi-square test.

## References

[1] Ahmadieh H., Moradian S., Faghihi H. (2005). Anatomic and visual outcomes of scleral buckling versus primary vitrectomy in pseudophakic and aphakic retinal detachment: six-month follow-up results of a single operation—report no. 1. *Ophthalmology*.

[2] Brazitikos P. D., Androudi S., Christen W. G., Stangos N. T. (2005). Primary pars plana vitrectomy versus scleral buckle surgery for the treatment of pseudophakic retinal detachment: a randomized clinical trial. *Retina*.

[3] Adelman R. A., Parnes A. J., Ducournau D., European Vitreo-Retinal Society (EVRS) Retinal Detachment Study Group (2013). Strategy for the management of uncomplicated retinal detachments: the European vitreo-retinal society retinal detachment study report 1. *Ophthalmology*.

[4] Heimann H., Bartz-Schmidt K. U., Bornfeld N. (2007). Scleral buckling versus primary vitrectomy in rhegmatogenous retinal detachment: a prospective randomized multicenter clinical study. *Ophthalmology*.

[5] Sodhi A., Leung L. S., Do D. V., Gower E. W., Schein O. D., Handa J. T. (2008). Recent trends in the management of rhegmatogenous retinal detachment. *Survey of Ophthalmology*.

[6] Figueroa M. S., Corte M. D., Sbordone S. (2002). Scleral buckling technique without retinopexy for treatment of rhegmatogeneous: a pilot study. *Retina*.

[7] D’Amico D. J. (2008). Clinical practice: primary retinal detachment. *The New England Journal of Medicine*.

[8] Lincoff H., Gieser R. (1971). Finding the retinal hole. *Archives of Ophthalmology*.

[9] Haug S. J., Jumper J. M., Johnson R. N., McDonald H. R., Fu A. D. (2016). Chandelier-assisted external subretinal fluid drainage in primary scleral buckling for treatment of rhegmatogenous retinal detachment. *Retina*.

[10] Ibrahim W. (2015). Twin-light-assisted scleral buckle for primary rhegmatogenous retinal detachment. *Egyptian Retina Journal*.

[11] Schwartz S. G., Kuhl D. P., McPherson A. R., Holz E. R., Mieler W. F. (2002). Twenty-year follow-up for scleral buckling. *Archives of Ophthalmology*.

[12] Criswick V. G., Brockhurst R. J. (1969). Retinal detachment: 360° scleral buckling as a primary procedure. *Archives of Ophthalmology*.

[13] Grizzard W. S., Hilton G. F., Hammer M. E., Taren D. (1994). A multivariate analysis of anatomic success of retinal detachments treated with scleral buckling. *Graefe's Archive for Clinical and Experimental Ophthalmology*.

[14] Shanmugam P. M., Singh T. P., Ramanjulu R., Rodrigues G., Reddy S. (2015). Sutureless scleral buckle in the management of rhegmatogenous retinal detachment. *Indian Journal of Ophthalmology*.

[15] Sternberg P., Tiedeman J., Prensky J. G. (1988). Sutureless scleral buckle for retinal detachment with thin sclera. *Retina*.

[16] Campochiaro P. A., Kaden I. H., Vidaurri-Leal J., Glaser B. M. (1985). Cryotherapy enhances intravitreal dispersion of viable retinal pigment epithelial cells. *Archives of Ophthalmology*.

[17] Haller J. A., Blair N., de Juan E Jr (1998). Transscleral diode laser retinopexy in retinal detachment surgery: results of a multicenter trial. *Retina*.

[18] Kita M., Fujii Y., Kawagoe N., Hama S. (2013). Scleral buckling with a noncontact wide-angle viewing system in the management of retinal detachment with undetected retinal break: a case report. *Clinical Ophthalmology*.

[19] Yokoyama T., Kanbayashi K., Yamaguchi T. (2015). Scleral buckling procedure with chandelier illumination for pediatric rhegmatogenous retinal detachment. *Clinical Ophthalmology*.

[20] Nam K. Y., Kim W. J., Jo Y. J., Kim J. Y. (2013). Scleral buckling technique using a 25-gauge chandelier endoilluminator. *Retina*.

[21] Jahangir T., Tayyab H., Naeem M., Lateef Q., Khan A. A. (2015). Modified scleral buckling with a non-contact wide angle viewing system and a 25g chandelier endoillumintor. *Annals of King Edward Medical University*.

[22] Seider M. I., Nomides R. E., Hahn P., Mruthyunjaya P., Mahmoud T. H. (2016). Scleral buckling with chandelier illumination. *Journal of Ophthalmic & Vision Research*.

[23] Aras C., Ucar D., Koytak A., Yetik H. (2012). Scleral buckling with a non-contact wide-angle viewing system. *Ophthalmologica*.

[24] Hu Y., Si S., Xu K. (2017). Outcomes of scleral buckling using chandelier endoillumination. *Acta Ophthalmologica*.

[25] Imai H., Tagami M., Azumi A. (2015). Scleral buckling for primary rhegmatogenous retinal detachment using noncontact wide-angle viewing system with a cannula-based 25 G chandelier endoilluminator. *Clinical Ophthalmology*.

[26] Tomita Y., Kurihara T., Uchida A. (2015). Wide-angle viewing system versus conventional indirect ophthalmoscopy for scleral buckling. *Scientific Reports*.

[27] Li X. J., Yang X. P., Lyu X. B. (2016). Comparison of scleral buckling using wide-angle viewing systems and indirect ophthalmoscope for rhegmatogenous retinal detachment. *International Journal of Ophthalmology*.

[28] Li H., Zhang Z., Liu X. (2016). 25-gauge chandelier illumination-assisted scleral buckling: a retrospective study. *Jacobs Journal of Ophthalmology*.

[29] Kapran Z., Uyar O. M., Bilgin B. A., Kaya V., Cilsim S., Eltutar K. (2001). Diode laser transscleral retinopexy in rhegmatogenous retinal detachment surgery. *European Journal of Ophthalmology*.

[30] de Miranda Gonçalves J. C., Farah M. E. (2004). Transscleral diode laser retinopexy in retinal reattachment surgery. *Arquivos Brasileiros de Oftalmologia*.

[31] Thompson J. T. (2008). The effects and action of scleral buckles in the treatment of retinal detachment. *Retina*.

[32] Williams G. A., Yanoff M., Ducker J. (2008). Scleral buckling surgery. *Yanoff and Ducker: Ophtalmology*.

[33] Lincoff H., Stopa M., Kreissig I. (2006). Cutting the encircling band. *Retina*.

[34] Sato E. A., Shinoda K., Inoue M., Ohtake Y., Kimura I. (2008). Reduced choroidal blood flow can induce visual field defect in open angle glaucoma patients without intraocular pressure elevation following encircling scleral buckling. *Retina*.

[35] Tsui I. (2012). Scleral buckle removal: indications and outcomes. *Survey of Ophthalmology*.

[36] Banaee T., Hosseini S. M., Helmi T., Ghooshkhanei H. (2015). Encircling narrow band versus buckle for retinal detachments with intrabasal or unseen retinal breaks. *Journal of Ophthalmic & Vision Research*.

[37] Kishore K., Peyman G. A. (2001). Chapter 8 Technique of scleral buckling for retinal detachment repair. *Vitreoretinal Surgical Technique, Second edition*.

[38] Yoshizumi M. O., Friberg T. (1983). Erosion of implants in retinal detachment surgery. *Annals of Ophthalmology*.

[39] Michels R. G. (1986). Scleral buckling methods for rhegmatogenous retinal detachment. *Retina*.

[40] Mazinani B. A., Rajendram A., Walter P., Roessler G. F. (2012). Does surgical experience have an effect on the success of retinal detachment surgery?. *Retina*.

[41] Kreissig I., Rose D., Jost B. (1992). Minimized surgery for retinal detachments with segmental buckling and nondrainage: an 11-year follow-up. *Retina*.

[42] Gogia V., Venkatesh P., Gupta S., Kakkar A., Garg S. (2014). Endoilluminator-assisted scleral buckling: our results. *Indian Journal of Ophthalmology*.

[43] Narayanan R., Tyagi M., Hussein A., Chhablani J., Apte R. S. (2016). Scleral buckling with wide-angled endoillumination as a surgical educational tool. *Retina*.

